# Management and Outcomes of Complications Related to BioZorb Use in Breast-Conserving Surgery: A Retrospective Single-Center Case Series Analysis

**DOI:** 10.31486/toj.25.0104

**Published:** 2026

**Authors:** Carolina Baz, Eve Paxton, Justin Turcotte, Meghan Milburn, Steven Woodward, Rubie Sue Jackson

**Affiliations:** ^1^ Department of Surgery, Luminis Health Anne Arundel Medical Center, Annapolis, MD; ^2^ The Rebecca Fortney Breast Center, Luminis Health Anne Arundel Medical Center, Annapolis, MD

**Keywords:** *Absorbable implants*, *breast*, *complications*, *fiducial markers*, *mastectomy–segmental*, *radiotherapy–adjuvant*, *radiotherapy planning–computer-assisted*

## Abstract

**Background:**

BioZorb (Hologic, Inc) is an implantable fiducial marker used in breast-conserving surgery. Since the US Food and Drug Administration (FDA) approved BioZorb in 2012, numerous adverse effects have been reported, prompting the FDA to issue a warning about potential risks on February 27, 2024. BioZorb was subsequently recalled, and on October 25, 2024, the FDA classified the recall as a Class 1, the most serious category, because of the risk of severe injury or death associated with the device. Limited research is available on the management of BioZorb-related complications.

**Methods:**

In a retrospective medical records review of 296 patients who had BioZorb placed and had more than 30 days of follow-up in the electronic medical record, we identified the patients who were treated for BioZorb complications from January 2012 to February 2025.

**Results:**

With an average follow-up of 38.4 months, 13 patients (4%) experienced device-related complications, identified at a median of 6.2 months. Most affected patients had multiple complications, with the most common being chronic pain (54%), surgical site infection (39%), and wound dehiscence (31%). BioZorb was removed in 62% of patients; the remainder of the patients were managed nonoperatively.

**Conclusion:**

We identified several complications associated with the use of BioZorb in our patient population, and most of the patients who experienced complications required device removal. Because complications can develop many years after the initial surgery, surgeons should be aware of the existence of complications and the varied presentations. To our knowledge, this study is the first report on the management of BioZorb complications and subsequent outcomes, providing valuable insights for surgeons.

## INTRODUCTION

Breast-conserving surgery followed by postoperative radiation is a well-established and often preferred option for patients with early-stage breast cancer, given the demonstrated equivalent or superior recurrence outcomes associated with this treatment compared to mastectomy.^1,2^ However, accurately identifying the tumor bed after breast-conserving surgery can be technically challenging.^3-5^ BioZorb (Hologic, Inc) is a bioabsorbable implantable tumor bed marker that facilitates targeted radiation therapy after breast-conserving surgery.^6,7^ After US Food and Drug Administration (FDA) approval in 2012, BioZorb was widely used to reduce tumor bed boost volumes.^8,9^ Despite evidence indicating favorable results in terms of improved cosmetic outcomes and decreased radiation exposure,^6,7^ several adverse effects related to the device were reported,^8,10,11^ prompting the FDA to release a communication on February 27, 2024, warning about the potential risk of serious complications.^12^ Later that year (October 25, 2024), BioZorb was recalled, and the FDA classified the recall as a Class 1, the most serious category, because of the risk for severe injury or death associated with the device.^12^

The current literature analyzing the most common adverse effects and their management is limited. Among the few studies available, most focus on short-term complications and have brief follow-up periods, which may have hindered the identification of long-term complications.^8,10,11^ Furthermore, these studies do not emphasize how the complications were managed or patient outcomes.

The purposes of this study were to identify the types of complications associated with BioZorb use in breast-conserving surgery, to discuss their management, and to examine patient outcomes.

## METHODS

Following institutional review board approval, we retrospectively identified patients treated at Anne Arundel Medical Center in Annapolis, Maryland, for BioZorb complications from January 2012 to February 2025. We used the search terms “implantable device,” “Hologic implant,” and “BioZorb” to identify patients. All complications, management, and outcomes available in the medical record were recorded. Data are presented as descriptive statistics and means. Analysis was done in SPSS Statistics, version 28 (IBM Corporation).

## RESULTS

We identified 296 patients who had BioZorb placed and had more than 30 days of follow-up in the electronic medical record. Of these 296 patients, 13 (4%) had a device-related complication documented. Patients had an average follow-up of 38.4 months. The median follow-up period during which complications were identified after device placement was 6.2 months.

Four patients (31%) experienced complications within 1 month after surgery, 4 patients (31%) between 1 month and 7 months, and 5 patients (39%) after 12 months (Table). Of the patients with complications after 12 months, 1 patient had a complication at 31 months, 2 at 36 months, 1 at 72 months, and 1 at 108 months following BioZorb placement.

**Table.** Follow-Up Periods, Complications, Management, and Outcomes Associated With BioZorb Placement

Patient	Time From Placement to Complication	Device Removed?	Time From First Complication to Removal	Time From Placement to Removal/Time From Removal To Last Clinical Follow-Up	Complications	Management	Outcomes
1	<1 month	Yes	2 months	2 months/32 months	Sinus tract in surgical wound Dehiscence	Debridement Device removal	Wound healed
2	<1 month	Yes	36 months	36 months/11 months	Chronic seroma Abscess	Percutaneous aspiration ×5 IV antibiotics Incision and drainage with device removal	Mastectomy with rotational flap, surgical site infection, wound debridement with areas of fat necrosis, left open, wound VAC, hyperbaric O_2_ therapy with Hydrofera (wound closing)
3	<1 month	Yes	6 months	6 months/36 months	Seroma Surgical site infection Dehiscence	Percutaneous aspiration ×2 Oral antibiotics Debridement Device removal	Developed nodules possibly indicating local recurrence Lost to follow-up
4	<1 month	Yes	3 days	1 month/62 months	Surgical site infection Dehiscence	Incision and drainage Oral antibiotics Device removal	Nonhealing wound after 3 months; additional debridement and tissue rearrangements by Plastic Surgery
5	1 month	No	NA	40 months	Surgical site infection Chronic pain	Percutaneous aspiration Oral antibiotics	Infection resolved BioZorb site still abnormally palpable
6	3 months	Yes	2 days	3 months/64 months	Dehiscence	Device removal	Nonhealing wound; tissue rearrangement and closure by Plastic Surgery
7	6 months	No	NA	74 months	Chronic pain Sensation of device movement	No specific treatment	Pain resolved 24 months after surgery
8	7 months	No	NA	43 months	Chronic pain	No specific treatment	Chronic pain
9	31 months	No	NA	43 months	Chronic pain	No specific treatment	Chronic pain
10	36 months	Yes	5 months	45 months/8 months	Surgical site infection Chronic pain	Oral antibiotics Device removal and debridement	Wound healed
11	36 months	Yes	2 months	41 months/37 months	Chronic pain Palpable mass^a^	Device removal	Pain resolved
12	72 months	Yes	15 days	69 months/5 months	Surgical site infection Abscess	Oral antibiotics Percutaneous drainage ×2 Incision and drainage with device removal	Cavitary wound left open followed by Plastic Surgery; plan for abdominal flap reconstruction of right breast
13	108 months	No	NA	105 months	Chronic pain	No specific treatment	Chronic pain

^a^During the reoperation, the device was found to have been reabsorbed despite complaints of palpable abnormality.

IV, intravenous; NA, not applicable; O_2_, oxygen; VAC, vacuum-assisted closure.

Chronic pain was the most common complication, reported by 7 patients (54%), followed by surgical site infection (5 patients, 39%), wound dehiscence (4 patients, 31%), abscess (2 patients, 15%), and seroma (2 patients, 15%) (Figure). Notably, 9 patients (69%) had more than 1 complication.

**Figure.**
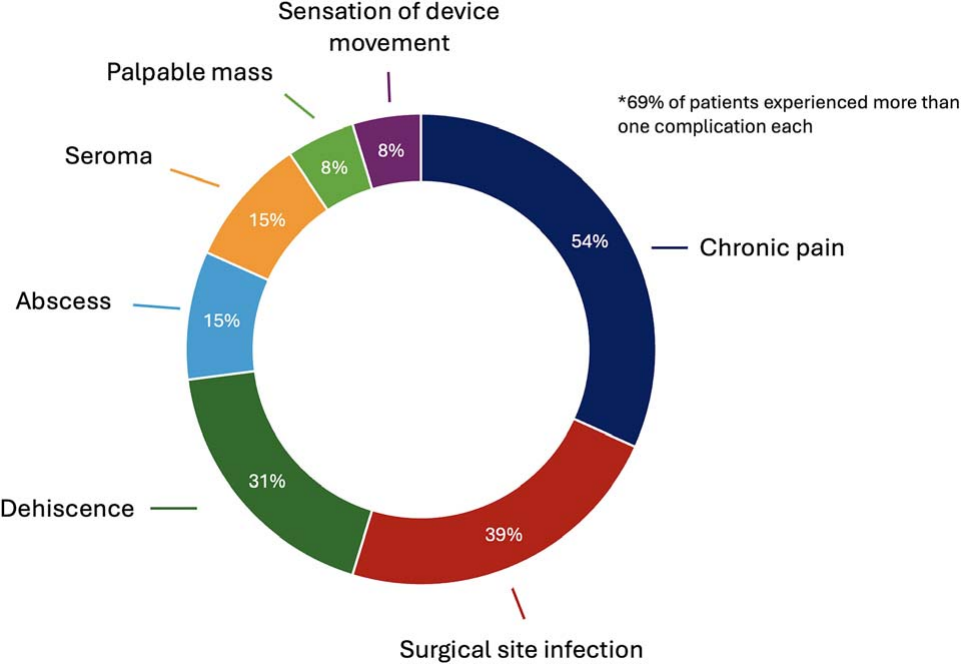
Complications associated with BioZorb placement.

Eight patients (62%) underwent BioZorb removal. Of these 8 patients, 4 (50%) experienced complications within the first month of placement, and 3 (38%) developed issues after 36 months. After removal, half (n=4) of the 8 patients had nonhealing wounds that required plastic surgery for further debridement, tissue rearrangements, and/or flap reconstruction.

Five of the patients with chronic pain were managed nonoperatively. Two patients experienced additional symptoms: 1 initially presented with a surgical site infection treated with oral antibiotics and percutaneous aspiration, while the other reported a sensation of device movement that did not require specific treatment. The 3 patients with chronic pain as their only complication needed no intervention. Among the 9 patients with multiple complications, most required antibiotics, aspiration, incision and drainage, or debridement. Seven of these 9 patients underwent device removal.

## DISCUSSION

Since the BioZorb device recall, published information on managing BioZorb-related complications has been limited. As noted in our case analysis, these complications can be complex and difficult to manage, with 23% of cases at our institution requiring plastic surgery for tissue rearrangements, closure, and flap reconstruction. Given the complexities of these issues, this case analysis provides insights into how these complications presented, how they were managed, and their final outcomes.

The few studies documenting complications associated with BioZorb focused on short-term outcomes and did not have follow-up periods exceeding 2 years.

Srour and Chung focused on 30-day complications in a study that included 89 patients.^10^ Superficial cellulitis was the most common complication, affecting 3 patients (3.3%), followed by abscess formation in 2 patients (2.2%).^10^ None of the patients required device removal, and they responded well to oral antibiotics. No patients experienced wound dehiscence, and only 1 had a symptomatic seroma that was managed conservatively. The median follow-up was 1.1 years, with a long-term complication involving 1 patient who experienced device migration from the breast to the axilla, necessitating surgical removal at 9 months postoperatively.^10^ In contrast, our study, with an average follow-up of more than 3 years and more than twice the number of patients, offers longer-term insights. Our larger cohort size and longer follow-up period likely explain the higher rate of serious complications, including surgery for device removal, as surgical intervention was usually needed approximately 12 months after BioZorb placement.

Kaufman et al prospectively followed 818 patients who underwent BioZorb placement after breast-conserving surgery and reported adverse effects in 3.8% of patients (n=31) within 30 days.^8^ Seroma was the most common complication, affecting 1.9% of patients, followed by breast infections and pain, both at 0.5%; hematomas at 0.2%; and poor wound healing and problematic palpability, both at 0.1%. The median follow-up was 18.2 months. Five of the 17 patients (29%) with infections that occurred throughout the entire follow-up period required device removal during the study; none required device removal within the first 30 days after placement.^8^ In contrast, in our study, 62% of patients who experienced complications required device removal. However, the retrospective nature of our study may have influenced these findings. Additionally, Kauffman et al reported pain in only 1 patient at 24 months.^8^ Our study, with a longer follow-up period, found that among the 7 patients who experienced pain after BioZorb placement, 3 continued to report chronic pain: 2 after 43 months and 1 after 105 months. Notably, in alignment with our findings, Kauffman et al indicated that some patients experienced multiple complications,^8^ suggesting this phenomenon may not be coincidental.

A case published in 2021 by Ju and Tsai reported a patient who developed several complications 7 months after device placement, including nipple discharge, erythema with purulent aspirate requiring antibiotic therapy twice, and ultimately BioZorb removal because of skin erosion.^11^ The experience of the patient reported by Ju and Tsai^11^ aligns with our finding that several patients experienced multiple serious complications.

The palpability of the device has also been a topic of interest in the limited publications addressing BioZorb complications. While complete reabsorption may take up to 1 year or longer, Srour and Chung reported that the device remained palpable in 63.6% of patients at a median of 1.1 years, with the longest follow-up period in that study being 2.8 years.^10^ In our study, 1 patient still had a palpable finding 3.6 years after BioZorb placement. Notably, in a reoperative case more than 2 years postsurgery, we found that the device had been reabsorbed, despite complaints of a palpable abnormality. The palpable findings in these cases correspond to indurated, abnormal tissue.

Our study has limitations. Because the study was retrospective, complications were recorded only if documented in a clinic follow-up note, thereby increasing the risk of selection bias, as patients may have presented to an outside hospital with complications that were not documented in our clinic. Another limitation is that our analysis includes patients treated at a single institution.

## CONCLUSION

To our knowledge, this study is the first to focus on managing complications related to BioZorb placement and patient outcomes, with the longest follow-up period to date—averaging 38.4 months—based on a retrospective review of 296 patients. Our study is also the first report to analyze complications in detail beyond 30 days after surgery. Four percent of patients in our population experienced a device-related complication. Chronic pain was the most common, followed by surgical site infection. Most adverse events required both conservative and invasive treatments, and 69% of patients had more than 1 complication. Most patients with complications required device removal at some point during follow-up.

## Notes

*This article meets the Accreditation Council for Graduate Medical Education and the American Board of Medical Specialties Maintenance of Certification competencies for Patient Care and Medical Knowledge.*
